# Supplementary material for: Selective PPARα Modulator Pemafibrate and Sodium-Glucose Cotransporter 2 Inhibitor Tofogliflozin Combination Treatment Improved Histopathology in Experimental Mice Model of Non-Alcoholic Steatohepatitis
Source: Cells. 2022 Feb 18;11(4):720. doi: 10.3390/cells11040720 (PMC8870369; doi:10.3390/cells11040720)
Supplement: Supplementary file 1 [file cells-11-00720-s001.zip › cells-1591248 author confirm sup .pdf]

Supplementary information

Selective PPARα modulator Pemafibrate and sodium-glucose cotransporter 2 (SGLT2) inhibitor Tofogliflozin combination treatment improves histopathology in experimental mice model of non-alcoholic steatohepatitis

Kentaro Murakami, Yusuke Sasaki, Masato Asahiyama, Wataru Yano, Toshiaki Takizawa, Wakana Kamiya, Yoshihiro Matsumura, Motonobu Anai, Tsuyoshi Osawa, Jean-Charles Fruchart, Jamila Fruchart-Najib, Hiroyuki Aburatani, Juro Sakai, Tatsuhiko Kodama and Toshiya Tanaka

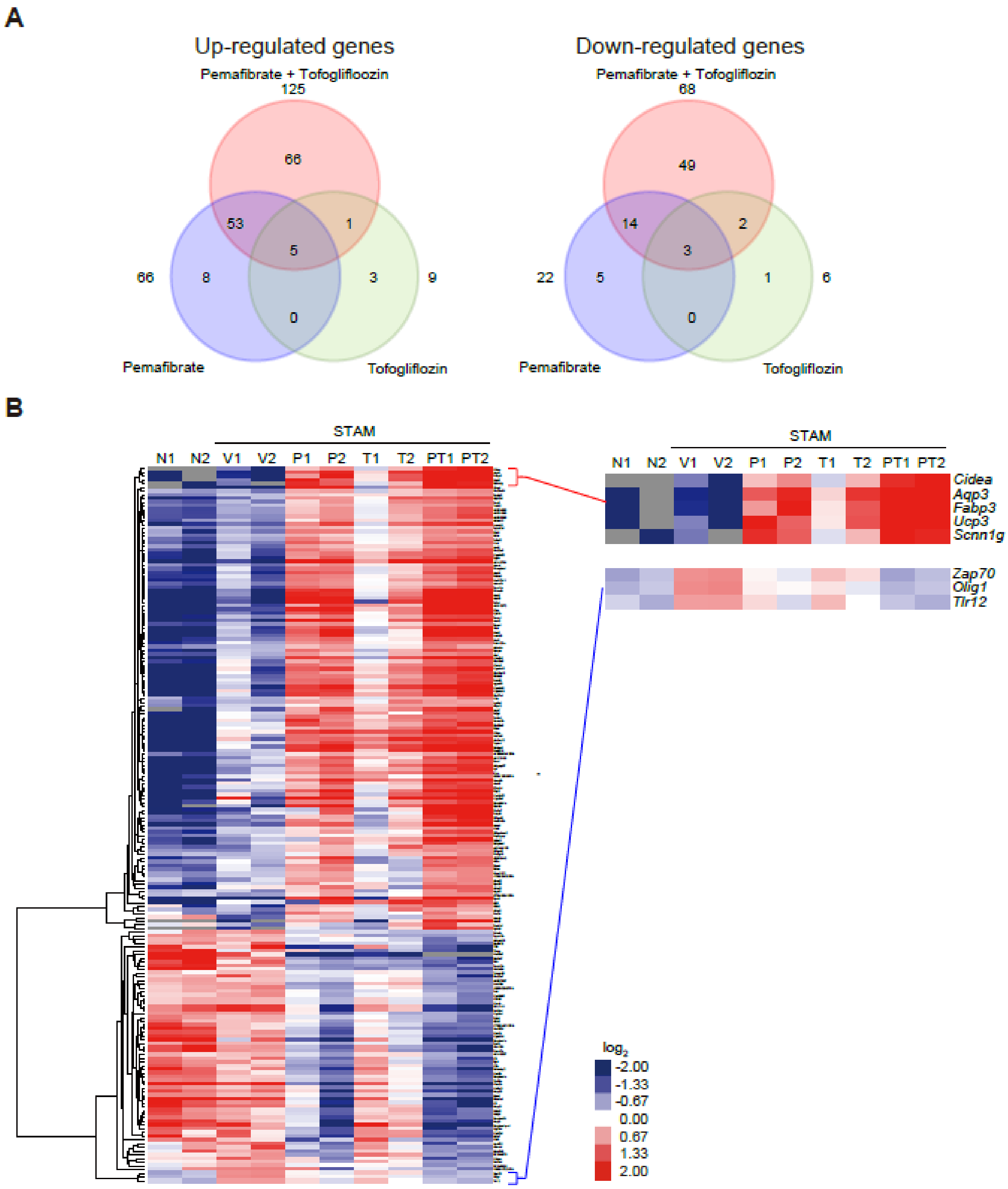

Figure S1. Pemafibrate and tofogliflozin combination regulated genes in STAM mouse liver. (A) Venn diagrams illustrating the overlap of either pemafibrate-, tofogliflozin-, and pemafibrate and tofogliflozin combination-treated STAM mouse liver. (B) Shown are genes whose transcript abundance is increased more than 2-fold or decreased greater than 2-fold in pemafibrate and tofogliflozin combination-treated STAM mice compared to control STAM mice group. N1 and N2: normal, C1 and C2: control, P1 and P2: pemafibrate, T1 and T2: tofogliflozin, PT1 and PT2: pemafibrate and tofogliflozin combination-treated group. For reference, a color intensity scale is included at the bottom.

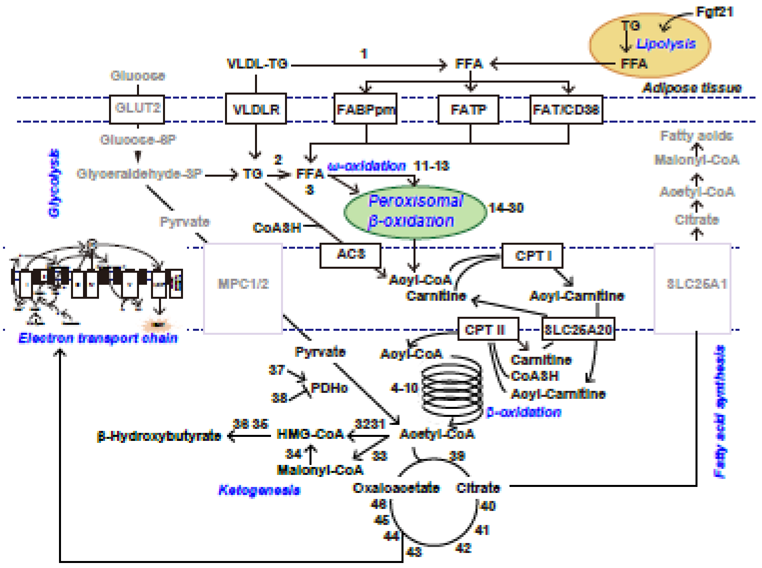

1. Lipoprotein lipase (Lpl)
2. Lipid lipase (Lipa, Lipc, Lipe, Lipg, Mgll)
3. Fatty acid binding protein (Fabp)
4. Acyl-CoA dehydrogenase (Acad)
5. Enoyl-CoA hydratase (Echs1)
6. Hydroxyacyl-CoA dehydrogenase (Hadh)
7. Mitochondrial 3-oxoacyl-CoA thiolase (Acaa2)
8. Enoyl-Coenzyme A delta isomerase 1 (Dci)
9. 2-4-dienoyl-Coenzyme A reductase (Decr1)
10. Hydroxysteroid 17-beta dehydrogenase 10 (Hsd17b10)
11. Cytochrome P450 family 4 (Cyp4a)
12. Alcohol dehydrogenase (Adh)
13. Aldehyde dehydrogenase (Aldh)
14. ATP-binding cassette, sub-family D (Abcd)
15. Phytanoyl-CoA hydroxylase (Phyh)
16. 2-hydroxyacyl-CoA lyase 1 (Hacl1)
17. Acyl-CoA oxidase (Acox)
18. Enoyl-CoA hydratase and 3-hydroxyacyl CoA dehydrogenase (Ehhadh)
19. Catalase (Cat)
20. Enoyl coenzyme A hydratase 1 (Ech1)
21. 2-4-dienoyl-Coenzyme A reductase (Decr2)
22. Enoyl-Coenzyme A delta isomerase 2 (Peci)
23. Hydroxysteroid 17-beta dehydrogenase 4 (Hsd17b4)
24. Acetyl-CoA acyltransferase 1 (Acaa1)
25. Carnitine acetyltransferase (Crat)
26. Carnitine O-octanoyltransferase (Crot)
27. Acyl-CoA thioesterase (Acot)
28. Nudix hydrolase (Nudt)
29. Peroxisomal membrane protein 2 (Pxmp2)
30. Solute carrier family 25 member 17 (Slc25a17)
31. Acetyl-Coenzyme A acetyltransferase 1 (Acat1)
32. HMG-CoA synthase 2 (Hmgcs2)
33. Acetyl-CoA carboxylase (Acac)
34. Acetoacetyl-CoA synthase (Aacs)
35. HMC-CoA lyase (Hmgcl)
36. D-beta-hydroxybutyrate dehydrogenase (Bdh)
37. Pyruvate dehyrogenase phosphatase (Pdp)
38. Pyruvate dehydrogenase kinase (Pdk)
39. Citrate synthase (Cs)
40. Aconitase 2 (Aco2)
41. Isocitrate dehydrogenase (Idh)
42. 2-Oxoglutarate Dehydrogenase Complex (Ogdh, Dlst, Dld)
43. Succinyl-CoA synthetase (Sucl)
44. Succinate dehydrogenase (Sdh)
45. Fumarate hydratase (Fh)
46. Malate dehydrogenase 2 (Mdh2)

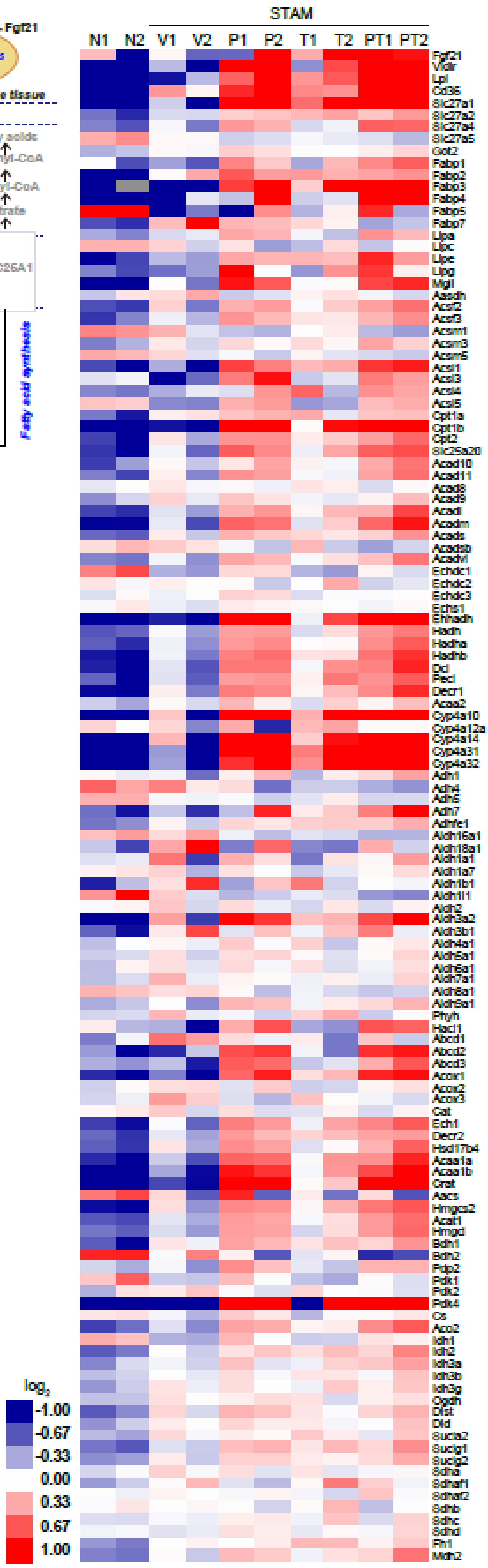

Figure S2. Effect of pemafibrate and tofogliflozin combination on fatty acid metabolism-related genes expression in STAM mouse liver. Schematic representation of the fatty acid metabolism pathways in the liver is shown in left panel. Heat

map showing changes in expression of selected fatty acid metabolism-related markers in normal (N1 and N2) or STAM control (C1 and C2) or pemafibrate-treated (P1 and P2) or tofogliflozin-treated (T1 and T2) or pemafibrate and tofogliflozin combination-treated (PT1 and PT2) STAM mouse liver.

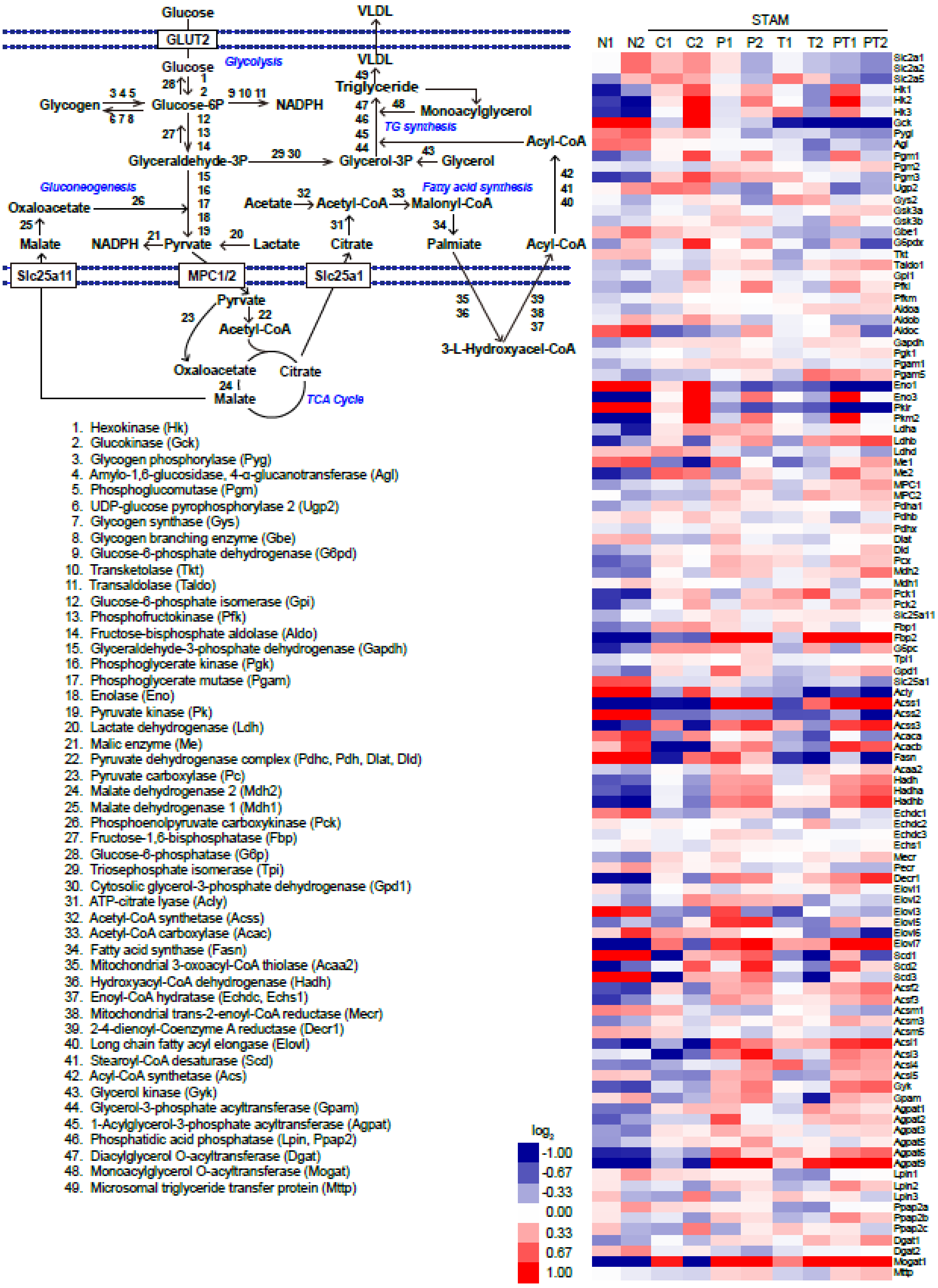

Figure S3. Effect of pemafibrate and tofogliflozin combination on glucose metabolism and triglyceride synthesis-related genes expression in STAM mouse liver. Schematic representation of the glycolytic, gluconeogenic, and triglyceride synthesis pathway in the liver is shown in left panel. Heat map showing changes in expression of selected glucose metabolism and triglyceride synthesis-related markers in normal (N1 and N2) or STAM control (C1 and C2) or pemafibrate-treated (P1 and P2) or tofogliflozin-treated (T1 and T2) or pemafibrate and tofogliflozin combination-treated (PT1 and PT2) STAM mouse liver.

Table S1. qPCR Primer lists.

Gene		Sequence
Slc2a2	Forward	5'- TGAGTTCCTTCCAGTTCGGC -3'
	Reverse	5'- AGCTTTCCGGTCATCCAGTG -3'
Hk1	Forward	5'- CAGTGTGAAGTCGGCCTGAT -3'
	Reverse	5'- GGCTCCCCATTCCGTGTTAA -3'
Gck	Forward	5'- CGGATGGTGGATGAGAGCTC -3'
	Reverse	5'- AGCACAAGTCGTACCAGCTC -3'
Gpi	Forward	5'- GCTTGTCCCCTGAGACTTCC -3'
	Reverse	5'- CGCTTCGAGAAACCACTCCT -3'
Pfk1	Forward	5'- GCACTGACATGACCATTGGC -3'
	Reverse	5'- CCGTCCCATCACCTCCAAAA -3'
Aldob	Forward	5'- CCACGAGACCCTCTACCAGA -3'
	Reverse	5'- TGTTTGTTCCTGCAAGCGGG -3'
Pck1	Forward	5'- TGCGGATCATGACTCGGATG -3'
	Reverse	5'- AGGCCCAGTTGTTGACCAAA -3'
Tpi	Forward	5'- CAGCAGGCACAGGAAGTACA -3'
	Reverse	5'- GCTCCAGTCACAGAACCTCC -3'
Gpd1	Forward	5'- GACCTGATGCAGACACCCAA -3'
	Reverse	5'- AAGCCAAGCCCATCACAGAA -3'
Gpam	Forward	5'- ACACAAGGCACAGAGGATGG -3'
	Reverse	5'- TTGCCTCTTGGACTCTGCTG -3'
Agpat6	Forward	5'- CCCTGCCATCTTTGGAGTGT -3'
	Reverse	5'- CCTCTCCTTGGCTCCTCTCT -3'
Agpat9	Forward	5'- TCCCTATCTGGCACCATCCA -3'
	Reverse	5'- GAGAGGTGTGATTGGCGACA -3'
Lpin2	Forward	5'- TTTCACGTACGCTTCGGGAA -3'
	Reverse	5'- TCCACAGCACTGCCATTGAT -3'
Ppap2b	Forward	5'- CAATTGCTCCGAGGGCTACA -3'
	Reverse	5'- AACATGGAGAAGGAGGCGTG -3'
Dgat1	Forward	5'- CTCAACTTTCCTCGGTCCCC -3'
	Reverse	5'- GATCAGCCCCACTTGAAGCT -3'
Mttp	Forward	5'- AGAGGACAGCTTTGTCACCG -3'
	Reverse	5'- CTTCCCGGGGATCATCCTTG -3'
Lipe	Forward	5'- TCCAGTTCACACCTGCCATC -3'
	Reverse	5'- GTCACACTGAGGCCTGTCTC -3'

Table S1. qPCR Primer lists (continued)

Gene		Sequence
Mgll	Forward	5'- ATCATCCCGGAGTCAGGACA -3'
	Reverse	5'- TTGGTCACTTCCGGAAGCTC -3'
Mogat1	Forward	5'- ACATCGTATCTCCACGTGGC -3'
	Reverse	5'- CTCCATCCTTGCTCAGCACA -3'
Vldlr	Forward	5'- TCAACTGCCCTTCTCGAACC -3'
	Reverse	5'- AGCCATCAACACAGTCTCGG -3'
Aqp9	Forward	5'- CGAGCCAAGAAGAACCTCGT -3'
	Reverse	5'- CCACCAGCCTTTTCTCGACT -3'
Gyk	Forward	5'- TGGCCTAATGAAAGCTGGGG -3'
	Reverse	5'- AGCACCCTGTTCCATACGTG -3'
Aldoa	Forward	5'- ACCCAGCAACAGACAGAGTT-3'
	Reverse	5'- CAGACAGCTCCTTCTTCTGC-3'
Slc2a5	Forward	5'- ATGGGTACAACGTAGCTGCC-3'
	Reverse	5'- GCGTCAAGGTGAAGGACTCA-3'
Khk	Forward	5'- GCTGGTGGTGCTGGACAT-3'
	Reverse	5'- AAGGAAAGGACAGTGCAGGA-3'

Dak	Forward	5'- TACGTTCTGACCTGGACACC-3'
	Reverse	5'- CTGCGATGACACCTGTTAGC-3'
Dgat2	Forward	5'- AAAGGATCTGCCCTGTCACG-3'
	Reverse	5'- CCTGCAGCTGTTTTTCCACC-3'
Perk	Forward	5'- TAGATGGACGAATCGCTGCA-3'
	Reverse	5'- GAGGAAGTTTTGTGGGTGCC-3'
Ire1a	Forward	5'- AGAAGATCCAGTCCTGCAGG-3'
	Reverse	5'- CCTTCGTTGTTCTTGCCTCC-3'
Atf6	Forward	5'- TGTCACTGGTCCTGGAAACA-3'
	Reverse	5'- GGCGCTGCTGGAAGTAATAA-3'
Grp78	Forward	5'- CGAGGAGGAGGACAAGAAGG-3'
	Reverse	5'- GTTGGCTATGATCTCCACGC-3'
Pdia1	Forward	5'- AGAGCAACTTCGAGGAGGC-3'
	Reverse	5'- CTTTGGCATACTCGGGAGC-3'
Nrf2	Forward	5'- TGCCCACATTCCCAAACAAG-3'
	Reverse	5'- GATGAGGGGCAGTGAAGACT-3'
Bak1	Forward	5'- TGCCTACGAACTCTTCACCA-3'
	Reverse	5'- GTAGACGTACAGGGCCAGAC-3'

Table S1. qPCR Primer lists (continued).

Gene		Sequence
Bax	Forward	5'- TCATGAAGACAGGGGCCTTT-3'
	Reverse	5'- AGACACTCGCTCAGCTTCTT-3'
Traf2	Forward	5'- ACTGTGCTGCCTGTGTCTAT-3'
	Reverse	5'- CCCTTCCAAGTGCATCCATC-3'
Xbp1	Forward	5'- GAAAGAAAGCCCGGATGAGC-3'
	Reverse	5'- CCATTCCCAAGCGTGTTCTT-3'
Phlda3	Forward	5'- CGCCACATCTACTTCACGC-3'
	Reverse	5'- TCTTGAACTTGACCAGGCCC-3'
Cide A	Forward	5'- CATACATCCAGCTCGCCCTT-3'
	Reverse	5'- CCAGGCCAGTTGTGATGACT-3'
Cide C	Forward	5'- TCATGGCTCACAGCTTGGAG-3'
	Reverse	5'- TCTGTCTCCACGATTGTGCC-3'
